# A homology independent sequence replacement strategy in human cells using a CRISPR nuclease

**DOI:** 10.1098/rsob.200283

**Published:** 2021-01-27

**Authors:** Eric Danner, Mikhail Lebedin, Kathrin de la Rosa, Ralf Kühn

**Affiliations:** Max Delbrück Center for Molecular Medicine of the Helmholtz Association, Berlin, Germany

**Keywords:** gene editing, CRISPR, exon replacement, replace editing

## Abstract

Precision genomic alterations largely rely on homology directed repair (HDR), but targeting without homology using the non-homologous end-joining (NHEJ) pathway has gained attention as a promising alternative. Previous studies demonstrated precise insertions formed by the ligation of donor DNA into a targeted genomic double-strand break in both dividing and non-dividing cells. Here, we demonstrate the use of NHEJ repair to *replace* genomic segments with donor sequences; we name this method ‘Replace’ editing (Rational end-joining protocol delivering a targeted sequence exchange). Using CRISPR/Cas9, we create two genomic breaks and ligate a donor sequence in-between. This exchange of a genomic for a donor sequence uses neither microhomology nor homology arms. We target four loci in cell lines and show successful exchange of exons in 16–54% of human cells. Using linear amplification methods and deep sequencing, we quantify the diversity of outcomes following Replace editing and profile the ligated interfaces. The ability to replace exons or other genomic sequences in cells not efficiently modified by HDR holds promise for both basic research and medicine.

## 1. Introduction

RNA-guided nucleases [1–3] have rapidly become foundational tools in facilitating genomic manipulations [4,5]. These nucleases target specific genomic loci and form a double-strand break (DSB). DNA repair processes are then leveraged to produce the desired outcome of the gene editing. Conventionally, specific genomic changes are made using homology directed repair (HDR) [6,7] with exogenously introduced DNA containing flanking sequences homologous to the targeted locus. One limitation of HDR-mediated genome editing is its restriction to the S/G2 phase, reducing or abolishing efficacy in slowly or non-dividing cells [8]. HDR, when used for gene editing, can be precise, but recent reports demonstrate greater error than often assumed, as incomplete or extraneous portions of the delivery vector can be copied into the genome [9–13]. On the other hand, the canonical non-homologous end-joining (NHEJ) pathway is traditionally viewed as error prone and relegated to disrupting gene function by inducing small insertions and deletions (InDels) during DSB repair. However, the high-fidelity aspects of NHEJ repair are often underappreciated as mutant InDels are easily observed, whereas non-mutagenic repair is indistinguishable from the original allele [14]. Furthermore, non-mutagenic repair by NHEJ reforms the Cas9 target site allowing for continued DSB formation. This may result in a final genomic population containing majority InDels despite NHEJ repair being predominately error-free.

Recently, increasing awareness of the fidelity and efficiency of NHEJ repair has led to the development of methods to produce genomic deletions and exogenous sequence insertions using this pathway. As NHEJ is highly active in all phases of the cell cycle, it has allowed precise edits in muscle cells and neurons [15–17]. Targeted deletions are produced by forming two DSBs with loss of the intervening sequence during repair. The ubiquitous nature of the NHEJ pathway allows for deletions in zygotes, as well as in adult tissue such as *in vivo* exon deletion in a mouse muscular dystrophy model [16,18]. Additionally, exogenously introduced dsDNA donor sequences can efficiently ligate into a single DSB by NHEJ (herein referred to as Insert targeting) [15,17,19–26]. With the NHEJ pathway conserved broadly, Insert targeting has been shown in plants [25], fish [19], cell lines [20–24,26], nondividing neurons and *in vivo* mouse tissues [15,17]. The ability to effectively integrate DNA across cell types has been used to tag genes with fluorophores [15,24,26], identify off-target CRISPR cleavage sites [27] and as a strategy for gene therapy by inserting functional coding sequences upstream of a disease causing exon [17].

Leveraging NHEJ repair to create large deletions and insert exogenous DNA posits the possibility of NHEJ-based sequence replacement; two DSBs are produced and a donor sequence without homology is ligated between the two breaks. This approach would enable the replacement of defective exons or regulatory sequences in a wide range of resting or dividing cells. NHEJ-based replacement has been demonstrated in plants, where HDR is often infeasible [28,29]. In order for NHEJ-based replacement to be considered a viable approach in human cells, demonstration of its efficiency and a thorough understanding of the editing outcomes is required. Here, we demonstrate efficient replacement of genomic sequences and exons with a donor sequence in human cells using NHEJ repair; we call this method Replace (Rational end-joining protocol delivering a targeted sequence exchange). Analysis of single-cell-derived clones provides conclusive evidence of Replace editing and efficiency. We further introduce sequencing pipelines for the precise quantification of the structural variants produced during Replace targeting and the InDels at the ligated interfaces. Together, our results and analysis strategies lay the groundwork for future applications of NHEJ-based Replace editing in gene therapy and research.

## 2. Results

**Figure 1.**
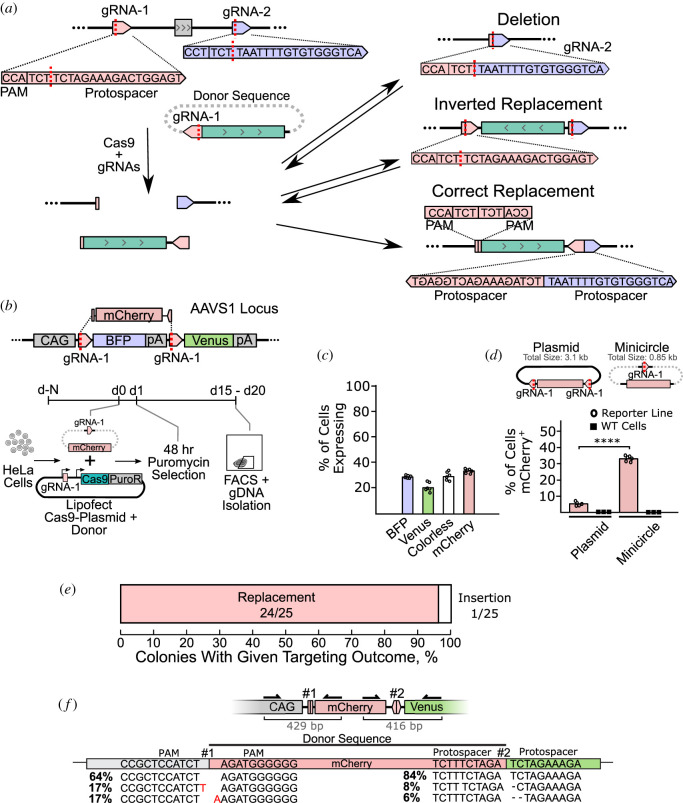
Replace targeting using HeLa reporter cells. (*a*) Overview of replace targeting concept: correct donor integration disrupts the Cas9 targeting sequence and is captured, while undesired products (deletion or inverted integrants) reform the gRNA target and can be further cut. (*b*) HeLa cells containing a fluorescent reporter system integrated into two AAVS1 loci. Targeting used gRNA-1 shown in (*a*) with two identical sites flanking BFP. Lipofection of Cas9-2A-puro/gRNA-1 and the donor sequence was followed by 48 h puromycin selection to ensure complete delivery. (*c*) Results of Replace targeting with a minicircle donor measured by FACS. BFP, original allele; Venus, deletion; mCherry, donor integration; colourless, allelic damage. Total sums to greater than 100% as cells can express two fluorophores. (*d*) Replace targeting using minicircles and plasmid sequence donors. WT cells are HeLa cells without the integrated reporter system. WT targeted identically. *p*-value was calculated using Student's *t*-test (****p-value <0.0001). (*e*) Single-cell sorted Replace targeted, mCherry^+^ cells were expanded and genotyped. Correct exchange of BFP by mCherry (Replacement) and mCherry integration flanking the BFP (Insertion) were both measured. (*f*) InDel frequencies at the interface of the integrated donor sequence in Replace targeted cells quantified by ICE deconvolution. Arrows represent primers for sequenced amplicons with sizes in grey.

Replace targeting (figure 1*a*) aims to exchange a genomic sequence with a double-stranded donor sequence without the use of homology. In this strategy, undesired products such as deletions or inverted donor sequences reform the Cas9 gRNA target sites and can be further targeted by Cas9, while the desired integration is captured. For initial validation, we used a fluorescence-based reporter system (figure 1*b*). The synthetic reporter system was created and integrated into two AAVS1 loci in a HeLa cell line. The reporter system contains a CAG promoter upstream of a BFP fluorophore. The BFP prevents the expression of a downstream Venus-pA. The cells initially are BFP^+^. Replace targeting exchanges the BFP cassette with a mCherry donor. Reporter HeLa cells were lipofected to deliver the donor sequence and Cas9 plasmid containing a puromycin resistance gene. Cells were selected for 48 h to ensure construct delivery and analysed after two weeks. Replacement targeting cleaved both sides of the BFP-pA cassette, with the excised sequence exchanged with the linearized mCherry donor sequence. Correct ligation of mCherry resulted in the loci expressing only mCherry. Deletion of the BFP cassette without replacement resulted in expression of the downstream Venus. Some alleles lost expression due to mutations or incorrect donor ligation.

Replace targeting of the reporter locus resulted in 34% mCherry^+^ cells (figure 1*c*). We compared the effect of delivering donor sequences within a plasmid or in the form of minicircles as a previous report showed minicircles to increase Insert efficiency [17] (figure 1*d*)*.* Minicircles are minimal plasmids and contain only the donor sequence and require only a single Cas9 DSB for linearization, whereas plasmids require two DSBs to excise the donor. Donor sequences delivered as minicircles resulted in a sixfold increase in cells with mCherry expression compared to plasmid delivery. We therefore used minicircles for Replace targeting in the remainder of this work. To address if mCherry expression was driven in part by off-target integration of the donor sequence, we Replace targeted, in an otherwise identical manner, wild-type HeLa cells. As these cells do not contain the AAVS1 integrated promoter and target site, only off-target integration could result in mCherry expression (figure 1*d*). Wild-type HeLa cells showed no mCherry expression indicating that the 34% mCherry^+^ cells in our original experiment are the result of correct integration at the target loci. mCherry^+^ cells were single-cell sorted, expanded and genotyped to check for correct sequence replacement. Twenty-four out of 25 analysed clones (i.e. 32% of all cells) contained the anticipated exchange of BFP with mCherry, while one clone contained an allele with mCherry insertion upstream of BFP (figure 1*e*). As HeLa reporter cells contained two copies of the reporter locus, we quantify the frequency of homozygous knock-in by simultaneously transfecting two donor sequences (mCherry and miRFP670) (electronic supplementary material, figure S1). By measuring the mCherry^+^, RFP^+^ and dual-positive populations, we calculated an average of 5% homozygous knock-in. Taken together, Replace targeting in our reporter system occurs as a major outcome, with a successful sequence exchange of at least one allele in 32% of cells.

During the ligation of the donor sequence into the genome, InDels may occur at the interface. To quantify short InDels, the gDNA of targeted and unsorted HeLa reporter cells was PCR amplified using primers flanking the ligated interface. The deconvolution of the Sanger traces of these amplicons provides an InDel estimate of the bulk population of Replace targeted cells (figure 1*f*). This analysis shows that short resection occurs in a minor (less than 16%) fraction of these small amplicons. The majority contained no InDel or a small, non-random insertion. Sanger sequencing of cloned individual alleles supports the bulk analysis (electronic supplementary material, figure S2A). The one or two nucleotide insertions were striking in that they matched the protospacer sequence downstream of the break site. It is known that SpyCas9 does not always form a canonical blunt end break three nucleotides downstream of the PAM, but can, at some frequency, form a staggered cut [30–33]. These non-random insertion InDels are probably caused by NHEJ acting on a Cas9-formed staggered cut (electronic supplementary material, figure S3). In this model, the sticky end cutting causes the PAM side of the break to contain extra nucleotides. These overhangs are filled during repair and appear as insertions when the two PAM sides are ligated in Replace targeting (figure 1*f*). This produces insertions in the interfaces of the PAM sides and not in ligated interfaces of two Protospacer sides of the break (electronic supplementary material, figure S3B).

**Figure 2.**
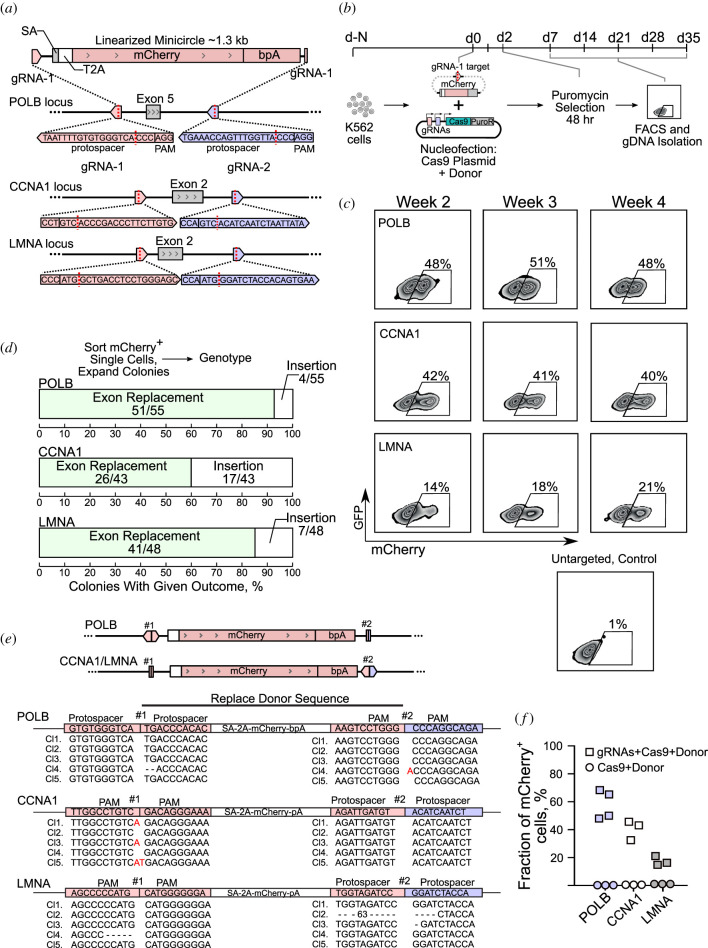
Exon Replace editing in K562 cells. (*a*) Replacement of *POLB* Exon 5, *CCNA* Exon 2 or *LMNA* Exon 2 with a cherry reporter including a splice acceptor (SA), T2A self-cleaving peptide and a polyadenylation site (bpA). (*b*) K562 cells cotransfected with Cas9-2A-puro/guides plasmid and mCherry Minicircles were puromycin selected for 48 h and followed by FACS analysis over five weeks. (*c*) mCherry^+^ count of transfected cell cultures was measured weekly by FACS. (*d*) Colonies of mCherry^+^ single cells were expanded and genotyped to check and quantify replacement of the exon with the donor sequence. (*e*) Sanger sequencing of amplicons showing InDels at the interface of the integrated donor sequence. (*f*) Biological replicates of Replace targeting of *POLB*, *CCNA1* and *LMNA* loci.

To test Replace targeting of an endogenous gene, we targeted three ubiquitously expressed loci in K562 cells: Polymerase Beta (*POLB)* exon 5, *CCNA1* exon 2 and *LMNA* exon 2. We replaced exons with a splice acceptor-2A-mCherry-pA donor sequence (figure 2*a,b*). Replace targeting resulted in reporter expression stable over weeks (figure 2*c*). Genotyping of mCherry^+^ single-cell derived colonies showed mCherry integration into the targeted locus in 100% of colonies. Correct replacement ranged from 60% to 93% of the colonies, but in some cells, the donor mCherry sequence inserted next to the original exon without replacing it (figure 2*d*). Sanger sequencing of the genome–donor sequence interface of individual PCR amplified alleles showed modest InDel formation in the correctly exchanged alleles (figure 2*e*; electronic supplementary material, figure S2). Replicate targeting experiments gave an average of 58%, 39% and 19% mCherry^+^ cells for *POLB* exon 5, *CCNA1* exon 2 and *LMNA* exon 2 respectively (figure 2*f*). All three targeted loci are triploid in K562 [34], assuming independence in the editing events, we can estimate the corresponding diploid cells would measure 44%, 28% and 13% mCherry^+^ for *POLB*, *CCNA1* and *LMNA*, respectively*.* Combining FACS and single-cell genotyping data allowed an estimate of 54% of *POLB*, 23% of *CCNA1* and 16% of *LMNA* Replace targeted cells with successful replacement in K562.

**Figure 3.**
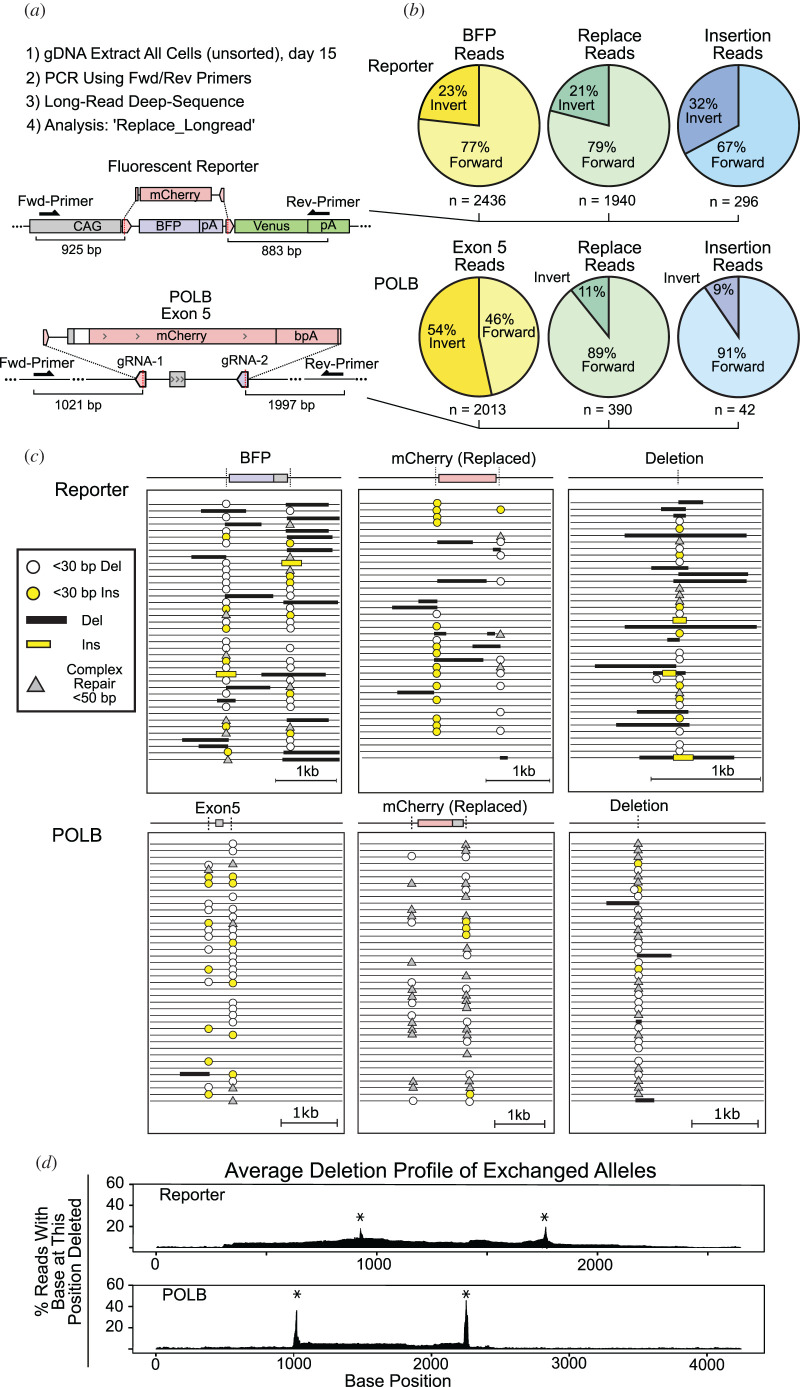
Long-read deep sequencing of Replace targeted cells. (*a*) Unsorted HeLa reporter cells or K562 *POLB* exon 5 targeted cells. Loci were amplified using the primers more than 800 bp from the Cas9 breaksites; amplicons were sequenced using PacBio technology (*b*). Directionality of structural variants formed after Replace targeting. BFP/exon 5 pie charts are alleles containing the original targeted allele and no donor. mCherry pie charts show alleles where mCherry replaced targeted sequence. Insertion is both donor sequence and targeted sequence in the loci. (*c*) Forty representative alignments of three major type of alleles: allele with original sequence, sequence exchanged with mCherry, or a deletion allele. (*d*) An average deletion profile of reads in which Replace editing using the mCherry donor was successful. Alignments were analysed for the fraction of reads containing a deletion at each base position. Asterisk denotes Cas9 cleavage site.

It is known that large-scale deletions may follow a single Cas9-driven DSB [35], and Replace targeting further complicates analysis due to the structural variants formed by the two genomic breaks and donor sequence integration. In order to quantify large deletions and the directionality of donor integration, we performed long-read deep-sequencing on amplicons of the targeted loci from unsorted Replace targeted HeLa cells and Replace targeted K562 cells (figure 3). We used primers 800–2000 bp away from the DSBs to generate long amplicons that were sequenced with PacBio technology. A bioinformatics pipeline was built to analyse large deletion and structural outcome frequencies (figure 3*a*) (electronic supplementary material, figure S4). While a donor sequence with no homology is expected to integrate equally in both directions, inspired by the work of Suzuki *et al*. [17], we designed a preferred orientation into our donor sequence without the use of homology (electronic supplementary material, figure S5). When the donor integrated in the undesired direction the ligated interface reform the Cas9 target site, whereas the desired orientation is unable to be further cut. Long-read deep sequencing measured the desired orientation of mCherry in 79% of reads where BFP was replaced in HeLa and 89% of alleles with *POLB* exon 5 replacement in K562 (figure 3*b*). Even alleles containing unintended donor insertion of mCherry into a DSB flanking the targeted sequence integrated preferentially in the designed orientation.

Alignment of the reads showed alleles with large-scale deletions (greater than 500 bp) occurred (figure 3*c*). Notably, individual reads showed that large-scale resection was frequently asymmetric with one side of the break undergoing dramatically larger resection. Viewing the frequency of a deletion at each base along the amplicon creates an averaged deletion profile and shows that the majority of loci experienced small-scale resection (figure 3*d*). Specifically, in successfully Replace targeted alleles, deletion mutations at the ligated junctions was smaller than 30 bp in greater than 90% of HeLa reporter reads, and smaller than 30 bp in greater than 95% of the *POLB* exon 5 reads. The ligated interface containing the protospacers were InDel-free in 79% of the correctly targeted reads of the HeLa reporter (electronic supplementary material, S1) and 63% InDel-free in the reads of the correctly targeted *POLB* K562 alleles, as measured by collapsing the long-read data (electronic supplementary material, S2).

**Figure 4.**
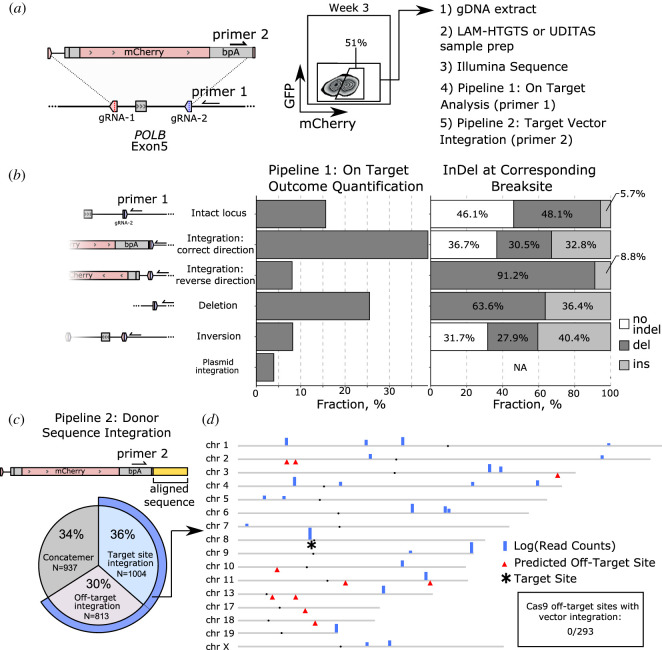
Linear amplification analysis of *POLB* exon 5; quantifying on-target Replace outcomes and mapping donor sequence integration. (*a*) Replace overview showing primer binding used for linear amplification. Unsorted gDNA was amplified with primer 1 or primer 2 and a universal primer, sequenced using Illumina technology, and analysed. (*b*) Pipeline 1 analysis of Uditas prepared samples quantifies outcomes at the targeted site with the corresponding InDels quantification. (*c*) Pipeline 2 analysis of UDiTaS prepared samples shows mCherry integration with an overall quantification of donor sequence integration. (*d*) Donor sequence integration mapped across the genome with read counts plotted logarithmically. Chromosomes with no integration sites were removed. Top 10 predicted Cas9 off-target sites shown with red triangles. The *POLB* locus on chromosome (chr) 8 is marked by an asterisk.

Linear PCR methods requiring only one gene-specific primer, such as UDiTaS [36] and LAM-HTGTS [37], offer more complete and quantitative measurements of DNA repair outcomes following a DSB. A gene-specific primer binds upstream of the targeted break site and a universal primer binding sequence is integrated downstream. Subsequently, the PCR amplifies the region across the break regardless of the structural variant, deletion size or translocation (figure 4*a*; electronic supplementary material, figure S4C,D). The UDiTaS method also contains a robust computational pipeline for CRISPR analysis. We modified this pipeline to extend the capabilities for Replace targeting with two pipelines (electronic supplementary material, figure S4E,F). Pipeline 1 closely follows the published UDiTaS pipeline; it aligns reads to the *in silico* reconstructed expected outcomes, performs InDel analysis and quantifies these measurements. The results of Pipeline 1 showed that at the targeted *POLB* locus donor sequence integrated in the preferred orientation at a 5 : 1 ratio to an inverted orientation (figure 4*a*,*b*). At 39% of all *POLB* alleles, the integration of the donor sequence in the desired orientation is the single most frequent outcome measured. Strikingly, more than one-third of these donors were integrated without an InDel formed at the ligated interface. This highlights both the efficiency and fidelity of Replace targeting for exon replacement.

As exogenously introduced DNA is known to integrate randomly into the genome [38], we developed Pipeline 2 to quantify and map the integration location of the donor sequence (electronic supplementary material, figure S4F). Using a primer that binds the donor sequence and points towards the ligated interface, we generated amplicons that contain the flanking genomic sequence. These amplicons were Illumina sequenced, and the genomic sequences beyond the end of the donor sequence were aligned to the human genome (figure 4*c*; electronic supplementary material, figures S4C and S5). Sequence alignment showed 55% on-target integration into the *POLB* locus. Thirty-four per cent of all measured donor sequences had formed concatenations; it remains to be determined where these concatenated sequences are integrating within the genome, but concatenation of exogenous dsDNA itself is a known phenomenon [9,39–42]. The donor sequences were shown to be integrated into the genome at more than 28 loci (figure 4*d*). Interestingly, none of the off-target integration mapped to any of the 293 predicted [43] SpyCas9 off-target sites.

## 3. Discussion

This work demonstrates that NHEJ-based genomic sequence exchanges are feasible and efficient in human cells. In the four loci tested, replacement was successful in 16–54% of cells; in one case, the desired product was the major outcome. We furthermore demonstrated targeted exon replacement via NHEJ in three widely expressed human genes. Based on the comprehensive analysis of our targeted alleles, we arrive at three design principles to guide future Replace work.

The first design aspect ensures the correct orientation of the donor sequence in the genome. Linearizing the donor sequence with the same gRNA that cuts the target locus allows incorrectly ligated donors to be re-cut and excised (electronic supplementary material, figure S6). It is crucial to add a gRNA targeting the sequence formed during a deletion. This gRNA re-opens alleles that form a deletion and also excises out incorrectly ligated donor sequences. The minimal requirement for this design is two gRNAs (electronic supplementary material, figure S6B,C). Long-read sequencing confirmed 89% of the donor sequences integrated in the designed orientation after *POLB* exon 5 Replace editing.

The second design principle is to avoid gRNAs that are involved in non-canonical SpyCas9 sticky end cutting. The frequency of ‘InDel free’ ligated interfaces measured in this work supports the idea that NHEJ repair is often not mutagenic [14]. We believe breaks introduced by Cas9 are often re-ligated to reform the original sequence, which can then be cleaved again—forming a break ligation cycle. This cycle continues until the Cas9 is no longer active or the target site forms an InDel during repair and disrupts Cas9 binding. For efficient Replace targeting, prolongation of this cycle provides more time to acquire and ligate the donor sequence in the correct orientation. InDel mutations remove alleles from the ligation cycle and thus decrease efficiency. One avoidable driver of InDel formation is non-canonical SpyCas9 cutting in which a staggered cut is formed [30–33]. The staggered cut is filled in and then ligated, duplicating the staggered nucleotide(s). The resulting small insertions are easily identifiable as they match the nucleotides of the protospacer sequence beyond the expected break site (electronic supplementary material, figure S3). Data from large gRNA screens suggest this mechanism as the predominant driver of +1 insertions [44]. The non-canonical cutting of SpyCas9 may be sequence or loci-dependent. Empirical testing of a gRNA by measuring InDel outcomes [45], therefore, allows us to avoid sites that incur staggered cuts.

The third concept is to design sacrificial sequences around the ligated regions to buffer possible resection and sequence deletions. While the overall rate of InDels and large-scale deletion is low, detrimental effects can be further reduced. During exon Replace targeting, we cut in intronic regions outside the splice site as short intronic InDels are less likely to be detrimental to gene function. Long-range deep sequencing showed that in our systems, the vast majority of the InDels are less than 30 bp long. Considering this, we recommend a sacrificial buffer 30 bp or greater be included on the flanks of the Replace construct to protect the splicing donor/acceptor and coding sequence. We currently use minicircles but also recommend such buffers on AAV delivered donor sequences too.

NHEJ-based sequence replacement has previously been explored using PCR fragments as donor sequence [46]. However, the genetic analysis in that study was not sufficient to distinguish successful replacement from other possible editing outcomes, such as unintended Insert targeting, structural rearrangements and off-target integration. Therefore, it remains to be confirmed and quantified in future work, if Replace editing with PCR donor templates is a viable strategy.

Measuring the outcomes of Replace targeting is complicated by the various structural rearrangements formed. Additionally, a growing body of literature documents complex outcomes following even simple Cas9-formed DSBs. These can include large-scale resection [35], chromosomal fusions [36], mis-spliced mRNA [47] and unintended vector integration into the break site [16]. In working towards a full understanding of the outcomes of Replace targeting, we developed multiple deep sequencing pipelines. Long-read sequencing of PCR amplicons of the targeted loci proved useful in illuminating resection profiles and gives insight into the orientation of the structural variants produced. However, samples prepared for long-read sequencing used two gene-specific primers and so suffered from PCR bias, over-representing the shorter amplicons, making quantitative comparisons of alleles of different lengths impossible. Traditional two primer PCR also requires both intact binding sites, and unable to amplify more complex repair products. To address these shortcomings, we turned to single primer amplification methods such as UDiTaS and LAM for quantitative analysis, as they amplify all outcomes approximately equally and measure more complex repair events. This allowed us to measure the frequency of deletions in *POLB* editing to be 26% of all alleles and only 16% of alleles maintained their wild-type allele. A total of 39% of alleles show correct integration of the donor, and the rest would not produce functional protein (structural inversions or deletions). This ability to measure knock-in and knock-out rates concurrently is helpful in understanding the function at the cellular level. In contrast with other studies measuring repair outcomes of a Cas9 DSB [36], we did not detect chromosomal fusions at our break points. However, this may be due to our analysis time point three weeks post-targeting, where alleles could have been selected out of the population. Beyond the utility for quantitative measurements on-target, these single-gene primer protocols are powerful for measuring unintended integration of introduced DNA sequences. For example, in treating a mouse model of muscular dystrophy, linear amplification measurements showed the therapeutic AAV unintentionally integrated into the Cas9 break site and throughout the genome [16]. These unintended integration of AAV in human cells may have a carcinogenic potential [48]. Others have recently demonstrated high rates of unintended on- and off-target integration of AAVs using single primer amplification [49]. Replace donor sequences have the potential to integrate into the target site or off-target into the genome. To our knowledge, this is the first work to map and quantify off-target integration or concatenation of donor sequences following NHEJ Insert or Replace targeting. Using a primer on the donor sequence, we detected substantial off-target integration of the donor. Strikingly, none of these off-target integration loci were within 5000 bases of the top 293 predicted Cas9 off-target sites. Rates of off-target integration may be similar for double-stranded HDR templates, but to our knowledge, off-target integration mapping by linear amplification has not been done after an HDR editing making comparison difficult. Single-stranded donor templates are known to integrate off-target less frequently [7,50], but off-target quantification has mainly relied on integration of large fluorescent cassettes and could benefit from using single primer amplification approaches.

There are currently over 3800 genes known to cause monogenic diseases with mutations often spread across multiple exons. Gene editing holds great potential for the treatment of such diseases, but reversing the genetic defects in terminally differentiated or resting cells remains a major challenge [51]. HDR is unable to target non-dividing cells [8], but the NHEJ pathway is known to be preserved across cells types and cycle [52]. NHEJ-based Insert targeting had previously been shown efficient in a wide variety of non-dividing and dividing cells *in vivo* and *in vitro.* The use of such NHEJ Replace editing holds the most potential for therapies looking to correct mutations in non-dividing cells by the replacement of exons. However, it was not clear if the NHEJ repair would allow for effective genetic replacement, but instead result in majority deletions, inserts or InDels. Additionally, the size variation between possible repair outcomes (i.e. deletions, insertions, replacements) makes their quantitative analysis challenging. In this work, we have demonstrated that the kinetics and fidelity of the NHEJ pathway allows for efficient replace targeting in human cells, and that a thorough understanding of the edited population can be achieved based on single primer PCRs, long PCRs and tailored analysis pipelines. While many questions, such as optimal donor delivery, remain to be addressed, our work provides the foundation for future applications of Replace editing for genome engineering.

## 4. Material and methods

### 4.1. Data and methods availability

Sequencing data are available. Sequence Read Archive (SRA) accession: PRJNA622521. Extended protocols are available at https://www.protocols.io/researchers/eric-danner/publications. Plasmids were submitted to Addgene (https://www.addgene.org/Ralf_Kuehn, #149344–#149354) and a folder of annotated genebank (.gb) files is added as electronic supplementary material, S1. All code used is available on Github: https://github.com/ericdanner. This includes scripts, Jupyter notebooks and Conda environments.

### 4.2. DNA constructs

Cas9-2A-puro targeting plasmid is Addgene ID 62988 with F1 sequence removed. The AAVS1 targeting fluorescent reporter system was modified from Addgene ID 60431. The neomycinR sequence was modified to a more robust form [53]. The RTTA3 gene was replaced by a BFP-pA-Venus-pA where the BFP is flanked by Rosa26 sequences constructed by Gibson Assembly. Guide RNA target sequences were ligated into BbsI cleaved plasmids using synthetic oligonucleotides (table 1). When more than one guide was necessary, the plasmids were combined using Gibson Assembly.

**Table 1.** Primer table.

name	sequence 5′ -> 3′
PacBio.Polb.F	ACTGGGGTTCAATTTTCTGTGTCCT
PacBio.Polb.R	GCTGTCATAGTGCCCATGTACAGAT
PacBio.HeLaReporter.F	GTTTCTTTTCTGTGGCTGCGTGAAA
PacBio.HeLaReporter.R	GGGGCTTCATGATGTCCCCATAATT
GenoPolb5_F	ccatacccggccATCTTTTAGA
GenoPolb5_R	ACTCCTTGATGATGGCCATGTT
GenoPolb3_F	CCACTCCCACTGTCCTTTCC
GenoPolb3_R	ATGCCCCATGCCATAAAGATGAGAG
GenoHeLaReporter_Replace5F	TTCGGCTTCTGGCGTGTGACC
GenoHeLaReporter_Replace5R	AAGGACAGCTTCAAGTAGTCGG
GenoHeLaReporter_Replace3F	GCGCCTACAACGTCAACATC
GenoHeLaReporter_Replace3R	GATCAGCTTCAGGGTCAGCTT
Tn5-Polb-Rev1	GCTTGAGGGCTTGTTCCAAATT
Tn5-Polb-RevNested	GTCTCGTGGGCTCGGAGATGTGTATAAGAGACAGACAAAAGAGGCCAAGCTGGAGCA
Tn5-Polb-mCherryfwd1	CCACTCCCACTGTCCTTTCC
Tn5-Polb-mCherryfwdNested	GTCTCGTGGGCTCGGAGATGTGTATAAGAGACAGgcaggagctcgtcgacccatg
Tn5-N501-tagmentationFwd	AATGATACGGCGACCACCGAGATCTACACTAGATCGCNNNNNNNNNNTCGTCGGCAGCGTCAGATGTGTATAAGAGACAG
Tn5-N501-tagmentationRev	[Phos]CTGTCTCTTATACA[ddC]
Tn5-universal_primerRev	AATGATACGGCGACCACCGAGATCTACAC
LAM-Polb-mCherryfwd1	[Btn]TGGAACAGTACGAACGCGCC
LAM-Polb-mCherryfwdNested	TCGTCGGCAGCGTCAGATGTGTATAAGAGACAGNNNNNTGCATCGCATTGTCTGAGTAGGT
LAM-Polb-mCherryrev1	[Btn]TGGTCACCTTCAGCTTGGCG
LAM-Polb-mCherryrevNested	TCGTCGGCAGCGTCAGATGTGTATAAGAGACAGNNNNNCCTCCACGTCACCGCATGTT
LAM-Adapter-Upper	GCGACTATAGGGCACGCGTGGNNNNNN[AmC3]
LAM-Adapter-Lower	[Phos]CCACGCGTGCCCTATAGTCGC[AmC3]
LAM-Adapter-U2C	GTCTCGTGGGCTCGGAGATGTGTATAAGAGACAGNNNNNGACTATAGGGCACGCGTGG

Minicircles are produced in engineered bacteria using arabinose-induced recombination to remove the plasmid backbone [54,55]. The ZYCY10P32T *E. coli* strain and the minicircle backbone were purchased from System Bioscience. After cloning in the sequence into the specific minicircle backbone, the plasmid is transformed into the ZYCY strain. The 200 ml culture was grown in TB media for 16 h. Then 200 µl of 20% l-arabinose was added and adjusted to pH 7 and 200 ml LB were added. The culture was then shaken at 32°C for 4 h to induce minicircle formation and slow cell division. An endotoxin-free purification kit (Macherey Nagel) was used following the protocol for low copy number plasmids. The resulting product contained plasmid and gDNA contamination. Restriction enzymes cutting the backbone and gDNA were added for 2 h. Then the resulting fragmented DNA was digested with PlasmidSafe DNase for 16 h (Epicure).

### 4.3. Cell culture and targeting

HeLa cells were cultured in DMEM, 10% FBS, 1% Penicillin/Streptomycin and passaged with trypsin every 3–4 days. To generate the fluorescent reporter line, plasmid #208 was cloned. Successful integration into the AAVS1 loci generated neomycin resistance. Cells were selected with 0.6 mg ml^−1^ G418 for one week. Single cells were FACS sorted into a 96-well plate and expanded. Colonies were checked for correct integration by genotyping and a clone with inserts on both alleles was expanded and used. Targeting of Reporter HeLa: 50 000 cells were reverse-transfected with 1.5 µg of Cas9_2A_puro/guide plasmid + 1.5 µg of MC or plasmid complexed with Lipofectamine 3000. The next morning 1.5 µg ml^−1^ puromycin was added for 48 h. Cells were then FACS analysed. mCherry^+^ cells were single cell sorted into a 96-well plate and expanded for genotyping. For the HDR targeting experiment, the guide RNA targeting the Insert site was used together with the donor plasmid.

K-565, a leukaemia cell line, were kept in IMDM, 10% FBS, 1% penicillin/streptomycin and split every 3 days. For targeting, cells were nucleofected using the Lonza 4D strips. A total of 5 × 10^5^ cells were resuspended in nucleofection buffer [56] with 1 µg Cas9/guide plasmid and 3 µg of minicircle and nucleofected using program FF-120. The following day puromycin (4 µg ml^−1^) was added for 48 h.

### 4.4. Genotyping

For single-cell clones or bulk sequencing, genomic DNA (gDNA) was extracted by quick extract (Lucigen). PCR amplification was performed with LongAmp Polymerase (NEB) or PrimerStar GXL (Takara). Primer pairs flanking the upstream cut site or downstream cut site were used. Amplicons were verified by gel extraction and Sanger sequencing. Amplicons from bulk sequencing were cloned into the TOPO vector (Invitrogen) before Sanger sequencing.

The frequency of homozygous and heterozygous integration in HeLa cells was determined by knocking-in mCherry and miRFP670 simultaneously. By measuring mCherry^+^, miRFP670^+^ and double positive cells, the homozygous knock-in could be calculated [7].

We used modified ICE analysis for deconvolution of amplicon Sanger trace data derived from unsorted Replace targeted cells [45]. The amplicons were made using a primer on the donor sequence and a primer on the genomic sequence flanking the ligated site. The amplicon was cloned into the TOPO vector and individual cloned alleles were Sanger sequenced along with the mixed PCR product. A cloned colony with Replace inserts without any InDels was identified, and these Sanger trace data were used as the ‘wild-type’ reference in ICE analysis.

### 4.5. Long-read deep sequencing and analysis

Bulk gDNA of targeted and control cells was amplified by PrimeStar GXL for Polb targeting. The HeLa synthetic reporter system required PCR with OneTaq (NEB) using the high GC content additive to amplify through the very GC-rich CAG sequence. Five-minute elongation steps were used to reduce PCR bias. Amplicons were cleaned by SPRI beads and quantified by Qubit. The Libraries were pooled and prepared for PacBio sequencing following company protocol. Data analysis was done using ‘Pipeline Longread’. This pipeline uses custom Python scripts for preprocessing and bins the reads into different structural variants: original exon, replacement, insertion or deletion. Alignments were done with BBmap or MiniMap2 [57] and visualized with IGV (Interactive Genome Viewer). Analysis of alignments was done in R using a modified script from (Github/pigX). Plotting was done in R or Python with a number of the plots included in the Jupyter Notebooks.

### 4.6. Uni-directional targeted sequencing sample preparation

Wild-type and treated cells having had the *POLB* exon 5 targeted showing 50% mCherry expression were used. Samples were prepared either as described in LAM-HTGTS [37] beginning with 500 ng of gDNA or based on the Tn5-Uditas protocol [36] beginning with 50 ng gDNA. LAM-HTGTS was done generally as published with a few modifications. A single biotinylated gene-specific primer was used to amplify 500 ng sonicated gDNA (1 kb peak) 80× rounds. Streptavidin Dynabeads were found to inhibit PCR so the concentration was reduced to 1/10th and used to capture the amplified sequence. Capture bead-DNA was washed and then the universal primer was ligated on the end. This adapter-ligated sequence was PCR amplified with a universal primer and a nested gene-specific primer 30×. We added Nextera adapters by 10× rounds of amplification. Gel extract 300–500 bp smear 300–500 bp, quantified by Qubit and Bioanalyzer, then sequenced with Illumina MiniSeq. For Tn5 sample preparation, we modified the UDiTaS protocol, 50 ng gDNA was washed 2× with SPRI beads. Tagmentation used hyperactive Tn5 produced by the Max Delbrueck Center protein production facility following published protocols [58]. Samples were tagmented to add the universal primer binding site. Sample was amplified with gene-specific primer and universal primer 15×. A nested primer with Illumina adapter sequences was added and followed by PCR 15×. Then Illumina adapters were added with 10× PCR. Amplicons 300–500 bp were gel extracted, quantified by Qubit and Bioanalzyer, then sequenced with Illumina MiniSeq.

### 4.7. Analysis of uni-directional targeted sequencing

All scripts and notebooks are on github.com/ericdanner/REPlacE_Analysis. The analysis of the linear amplified sequences was based on the Uditas software (https://github.com/editasmedicine/uditas). De-multiplexed samples are run through pipeline 1 or pipeline 2. Pipeline 1 generates amplicons of the various expected outputs and does a global alignment using Bowtie2 [59]. Reads that align well and cover the ligated junctions are analysed for InDels. If the samples were prepared with Tn5, they contained UMIs. Unique UMIs are tallied and editing outcomes are quantified. LAM samples do not contain UMIs. In Pipeline 2, the reads are checked for correct on-target priming. The samples are then trimmed using Cutadapt [60] up to the expected break site leaving only the sequence downstream of the break site. This sequence is aligned globally using Bowtie2 to an index file containing hg38 and the targeting vector.

## Supplementary Material

Long-Read HeLa Reporter analysis

## Supplementary Material

Long-Read K562 POLB analysis

## Supplementary Material

Annotated genebank files

## Data accessibility

Sequencing data are available. Sequence Read Archive (SRA) accession: PRJNA622521. Extended protocols are available: https://www.protocols.io/researchers/eric-danner/publications. Plasmids were submitted to Addgene: https://www.addgene.org/Ralf_Kuehn (#149344–#149354) and a folder of annotated genebank (.gb) files is added as electronic supplementary material, S1. All code used is available on Github: https://github.com/ericdanner. This includes scripts, Jupyter notebooks and Conda environments.
